# Decision-making in shoaling: zebrafish integrate cues of familiarity and group size

**DOI:** 10.1007/s10071-025-02008-2

**Published:** 2025-11-11

**Authors:** William T. Swaney, Amy Jose, Chelsie Hirons-Major, Adam R. Reddon

**Affiliations:** School of Biological and Environmental Sciences, Liverpool John Moores University, Byrom Street, Liverpool, L3 3AF UK

**Keywords:** Shoaling behaviour, Decision-making, Familiarity, Group size, Numerosity, *Danio rerio*

## Abstract

Social groups vary in the benefits that they offer to individuals through characteristics such as group size and composition, and consequently individual animals often exhibit preferences for groups with properties indicating greater benefits for members. Animals choosing between social groups may have to balance different preferences and integrate information about multiple group features to make optimal decisions and select the group that offers the greatest net benefit. We investigated how preferences for familiar individuals and for larger groups interact in the decision-making of zebrafish (*Danio rerio*) given a choice between two conspecific shoals. Adult subjects were given a series of choice tests with a shoal of four familiar fish, and a shoal of between four to eight unfamiliar fish. Subjects were tested on their preferences to spend time in proximity to the two shoals, and to interact with them. Zebrafish preferred to interact with the familiar shoal when both shoals comprised four individuals, however they did not show a preference for either shoal when choosing between four familiar fish and either five or six unfamiliar fish. When choosing between four familiar fish and either seven or eight unfamiliar fish, zebrafish showed clear preferences for the larger unfamiliar shoals over the familiar shoals. Our findings establish that zebrafish evaluate both the size and familiarity status of conspecific shoals, and integrate these multiple sources of information into social decision-making.

## Introduction

The benefits of group living for individuals likely explain its prevalence among animals (Ward and Webster 2016), despite the associated costs. Across taxa, group living provides key benefits such as reduced predation risk (Wrona and Dixon 1991; Polyakov et al. 2022), enhanced foraging efficiency (Cvikel et al. 2015), improved mating opportunities (Lindström and Ranta 1993; Ebensperger et al. 2019), and better offspring care (Feeney et al. 2013). Since these benefits and associated costs vary with group characteristics (e.g. size, composition), individuals are expected to exhibit group preferences and actively choose groups that maximize net benefits. However, different groups will often differ on multiple dimensions linked to benefits and costs for members and so when choosing between social groups, individuals may need to evaluate disparate information cues to make optimal decisions over which to join (Budaev et al. 2019). The integration of multiple sources of information in animal decision-making can involve information gathered via multiple sensory modalities (Taylor and Ryan 2013; Ota et al. 2015), or the evaluation of distinct cues of a single sensory type to reach a decision (Clemens et al. 2014; Sharp et al. 2015). Although there is empirical evidence of such integration of information cues in grouping decisions (Ward and Mehner 2010), there are outstanding questions regarding which group properties animals evaluate in social choices, and how they integrate conflicting or ambiguous cues.

In many shoaling and schooling fish species, individuals exhibit social preferences for shoals exhibiting properties linked to socially derived benefits for the individual. Zebrafish (*Danio rerio*) prefer well-fed conspecifics over hungry ones (Krause et al. 1999), while guppies (*Poecilia reticulata*) prefer shoals that have received foraging training over naïve shoals (Lachlan et al. 1998), suggesting discrimination of shoals based on cues linked to foraging. Preferences associated with mating are also common, for example three-spined sticklebacks (*Gasterosteus aculeatus*) and guppies are more attracted to shoals that offer greater potential mating opportunities (Webster and Laland 2013; Rystrom et al. 2018). Shoal size has been shown to be negatively associated with predation risk (Ioannou et al. 2011; Polyakov et al. 2022; Pacher et al. 2025), and preferences for larger shoals are seen in many species (Wong and Rosenthal 2005; Mehlis et al. 2015; Seguin and Gerlai 2017). Many fish also prefer shoals of phenotypically similar individuals (Rosenthal and Ryan 2005; Rodgers et al. 2010; Cattelan and Griggio 2018), which is thought to reduce an individual’s risk of predation through the ‘oddity effect’ (Landeau and Terborgh 1986). Preferences for familiar individuals over unfamiliar ones have also been demonstrated in many species (Magurran et al. 1994; Jordan et al. 2010; Swaney et al. 2024), and these widespread preferences for familiar shoals appear be linked to predation and foraging benefits for individuals. Familiar shoals have been shown to detect and escape from predators faster (Griffiths et al. 2004; Nadler et al. 2021), to engage in more effective defensive signalling (Bairos-Novak et al. 2019), and to shoal more cohesively (Chivers et al. 1995; Lucon-Xiccato et al. 2022), leading to reduced risk of predator attack (Ioannou et al. 2012). Individuals in familiar shoals also locate food faster (Ward and Hart 2005), and feed at higher frequency (Griffiths et al. 2004) than in unfamiliar shoals. There is less aggression between competing individuals in familiar shoals (Utne-Palm and Hart 2000), an effect that may point to the social dynamics that underlie familiarity effects. Familiarity is only established after approximately two weeks in guppies (Griffiths and Magurran 1997) and three-spined sticklebacks (Utne-Palm and Hart 2000), time that may be necessary for learned individual recognition of group members, and stabilisation of intra-group dominance relationships (Höjesjö et al. 1998).

While many studies of shoaling preferences in social fish focus on a single property of groups, natural shoals are more likely to differ in multiple ways that may influence shoaling decisions, for example shoal size, sex ratio, familiarity, phenotypic homogeneity etc. An individual choosing between shoals may have to assess multiple group properties to choose the most advantageous shoal to join. Shoaling decisions when choosing between groups with competing differences will depend on how the groups differ, and how large those differences are. Individuals may prioritise one group property over others, or they may integrate information about different group properties to reach a decision. Male guppies exhibit preferences for larger shoals over smaller ones (Lucon-Xiccato et al. 2016), and also prefer groups of females to males. However, if choosing between a smaller group with more females and a larger group with fewer females, they prefer to associate with the larger group (Lindström and Ranta 1993), suggesting they prioritise the anti-predation benefits of a larger group over preferences for the opposite sex. Three-spined sticklebacks’ shoaling decisions are influenced by both the size and the density of conspecific shoals: choice tests with groups that vary across both these dimensions have shown that individuals do not prioritise one property over the other, but integrate both to make shoaling decisions (Frommen et al. 2009). Alternatively, competing preferences may result in no clear preference if group properties balance each other out, or they may result in a random choice if individuals are unable to decide. Preferences for larger shoals, and for shoals that are phenotypically similar to the choosing individuals have been demonstrated in both swordtails (*Xiphophorus birchmanni x X. malinche*; Wong and Rosenthal 2005) and fighting fish (*Betta splendens*; Blakeslee et al. 2009), however when these preferences conflict in choice tests with large, dissimilar shoals versus small, similar ones, neither species shows a consistent preference but appear to choose at random.

We were interested in how competing preferences influence social decision-making, and how individual fish reach a decision when choosing between groups that differ in more than one dimension. Working with zebrafish which exhibit robust shoaling behaviour and are tractable for laboratory study (Spence et al. 2008), we focused on their preferences for larger shoals (Seguin and Gerlai 2017), and for familiar shoals (Swaney et al. 2024). We investigated how individual zebrafish would decide between groups that varied along both of these dimensions through a series of shoaling choice tests with familiar and unfamiliar shoals. By keeping the size of the familiar shoal constant in these tests but varying the size of the unfamiliar shoal from equivalent, to double that of the familiar shoal, we sought to test how individual zebrafish would prioritise shoal size and shoal familiarity. If either familiarity or shoal size is more important for zebrafish, we predicted that subjects would always choose on the basis of one of these preferences and ignore the other group property. If the conflicting preferences for familiarity and shoal size cannot be resolved by zebrafish, then we expected subjects to choose randomly in all tests when the unfamiliar shoals were larger than the familiar shoals. If zebrafish can integrate these preferences, we predicted that subjects would prefer familiar shoals when both shoals were of equal size, would prefer the largest unfamiliar shoals over familiar shoals, and would exhibit intermediate preferences in trials when the unfamiliar shoals were only somewhat larger that the familiar shoals.

## Methods

### Animals

The study involved 12-month old, wild type AB strain male and female zebrafish from our stock population, originally established with larvae from multiple clutches obtained from the University of Manchester in 2017 and 2018. Six months prior to the start of experiments, we randomly selected 67 individuals from stock groups and moved them into 45 × 30 × 30 cm LxWxH glass aquarium tanks to form five new, mixed-sex groups of between 11 and 16 experimental subjects. A further 20 mixed-sex individuals were randomly selected to be used as unfamiliar stimulus fish and moved into a 60 × 30 × 38 cm LxWxH glass aquarium tank. Each home tank contained 2 cm gravel, three plastic plants, a foam bubble filter, a floating thermometer and a heater, and were blacked out on three sides so that fish could not see into other tanks. Water was maintained at 27 ± 1 °C, lights were on a 12 H:12 H light: dark cycle, and fish were fed daily with Tetramin Flakes fish food.

### Experimental design

Individual subjects were given a series of two-choice shoaling tests with a shoal of familiar fish from their own home tank, and a shoal of unfamiliar fish from the unfamiliar stimulus tank. Subjects experienced five different versions of the shoaling tests: the size of the unfamiliar shoal consisted of either 4, 5, 6, 7 or 8 individuals, while the familiar shoal always consisted of 4 individuals. Subjects from each home tank experienced each version of the test only once, and the order of testing with each version was randomised for each home tank group to control for order effects. All subjects from a single home tank were tested in a single day on one test version, and there was an interval of at least 3 days between tests for fish from any single home tank.

### Behavioural tests

**Fig. 1:**
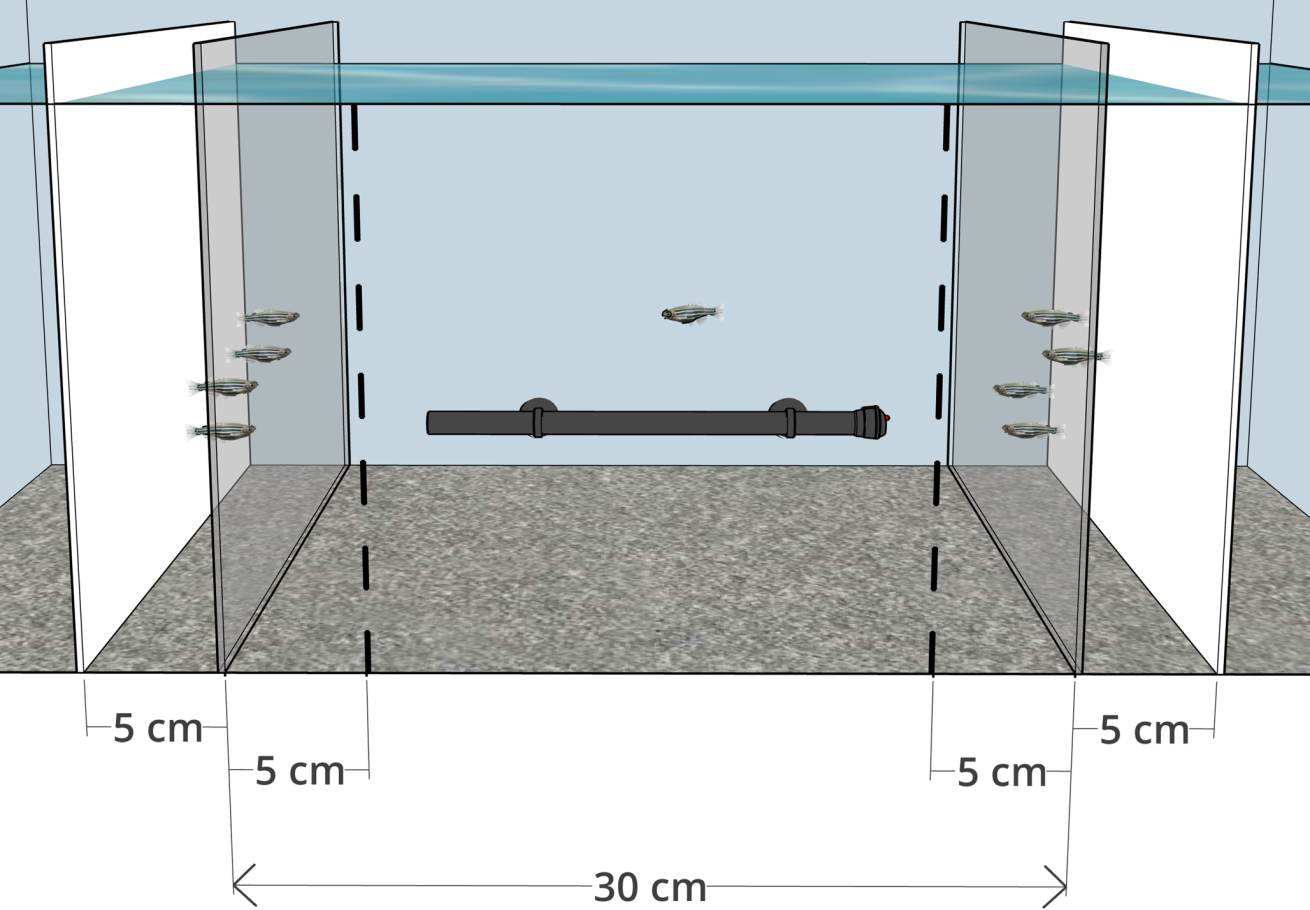
A schematic of the test tank, showing a single focal zebrafish subject in the central 30 cm area, with familiar and unfamiliar stimulus zebrafish behind transparent partitions at each end, and opaque partitions behind them forming the holding areas for the stimulus fish. The familiar shoal always consisted of four individuals, while the unfamiliar shoal consisted of either 4, 5, 6, 7 or 8 individuals. Dotted lines mark the designated 5 cm shoaling zones in front of the transparent partitions. Subjects were scored on the time they spent attempting to interact with the shoals at the transparent partitions, and the time spent within the 5 cm shoaling zones. Figure created using SketchUp (Trimble Inc.)

Testing took place in a 60 × 30 × 38 cm LxWxH glass aquarium tank containing 20 cm depth of water at 27 ± 1°C, a water heater and gravel substrate. Clear plastic partitions were used to create a 30 cm-wide central area for the focal subject. White plastic partitions were placed beyond each end to create two 5 cm-wide holding areas for the familiar and unfamiliar shoals (Fig. 1). Shoaling zones for the focal subject were defined by marks on the front of the tank, 5 cm from the clear partitions (equivalent to two body lengths). Male and female zebrafish do not exhibit significant preferences for shoals of either sex (Snekser and Diestler 2023), nor for single sex shoals over mixed-sex shoals (Ruhl and McRobert 2005), and we therefore used both male and female zebrafish in all stimulus shoals. Prior to testing, two males and two females from one of the home tanks were randomly selected and transferred with a net into one of the holding areas to serve as the familiar shoal, while 2–4 males and 2–4 females were selected from the unfamiliar aquarium and transferred into the other holding area to serve as the unfamiliar shoal. Fish for the unfamiliar shoal were chosen to be of similar size to those selected for the familiar shoal, based on visual inspection. The side on which the familiar and unfamiliar shoals were presented was randomised and switched every four tests, and stimulus fish were allowed to habituate for five minutes before testing was started, and after switching sides.

For each test, a rectangular 15 × 12 × 25 cm LxWxH transparent plastic tube was placed in the middle of the central focal area, a subject was selected at random from the home tank being tested and transferred directly from their home tank into the tube. After two minutes of habituation, the tube was raised and removed, to release the subject and start the 10-minute test. The subject’s behaviour was then scored live by an observer using JWatcher (https://www.jwatcher.ucla.edu) to record subject behaviour. Two aspects of subject behaviour were recorded: time spent in the 5 cm shoaling zones in front of each stimulus shoal, and time spent ‘interacting’ with each stimulus shoal, defined following Lindeyer et al. (2015) as when subjects were actively swimming head-first against the transparent partition towards the stimulus fish. Subjects were scored as inside or outside a shoaling zone when at least the front half of the body had crossed the marked boundary lines.

### Data and statistical analysis

For each subject, the data from JWatcher was used to calculate an interaction preference score (time interacting with the familiar shoal divided by total time interacting with both shoals), and a shoaling preference score (time spent in the familiar shoaling zone divided by total time in both shoaling zones). These preference scores were then separately analysed for each version of the two-choice shoaling tests (4, 5, 6, 7, or 8 fish in the unfamiliar stimulus shoal), using one-sample t-tests comparing the scores against a hypothetical mean of 0.5, which would indicate an equal preference for each shoal. Data for all tests met assumptions of normality of data and absence of outliers, as confirmed by visual inspection of QQ plots and boxplots. A Bonferroni-Holm correction was applied to the p-values of the ten separate analyses to control for the repeated testing of subjects. R v.4.5.0 (R Core Team 2025) and RStudio v.2025.05.1 (Posit Team 2025) were used to analyse all data and to create figure plots. Data and code for the analyses will be available on publication at Zenodo (10.5281/zenodo.15625544).

## Results

**Fig. 2:**
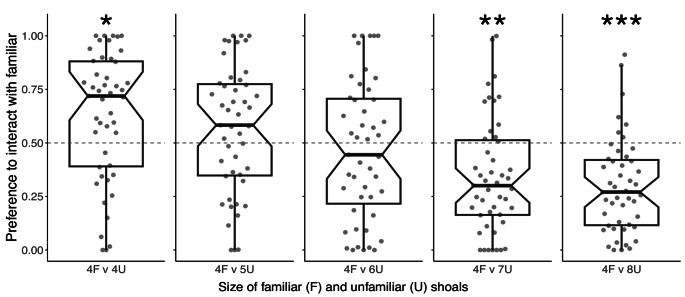
Notched box and whisker plots with overlaid raw data showing proportional time spent interacting with familiar stimulus fish relative to total time interacting with both familiar and unfamiliar stimulus fish. Values of > 0.5 indicate that subjects spent more time interacting with familiar fish, while values of < 0.5 indicate that subjects spent more time interacting with unfamiliar fish. Central lines represent medians, boxes extend from the 1st to the 3rd quartile, whiskers show the range up to 1.5x beyond the boxes, and the notches indicate approximate 95% confidence intervals for the medians

Subjects’ interaction preferences varied according to the number of unfamiliar stimulus fish in the tests (Fig. 2). They interacted significantly more with familiar than unfamiliar fish when both shoals consisted of 4 fish (mean ± SE interaction preference score = 0.630 ± 0.044, t_46_ = 2.959, *p* = 0.029), but did not show significant preferences for either shoal based on interaction behaviour in the tests with 5 unfamiliar fish (0.564 ± 0.043, t_46_ = 1.480, *p* = 0.437), or 6 unfamiliar fish (mean = 0.467 ± 0.047, t_46_=−0.700, *p* = 0.978). Subjects interacted significantly more with the larger unfamiliar shoal in tests with 7 unfamiliar fish (mean = 0.342 ± 0.039, t_46_=−4.073, *p* = 0.001), and 8 unfamiliar fish (mean = 0.298 ± 0.032, t_46_=−6.264, *p* < 0.001).

**Fig. 3:**
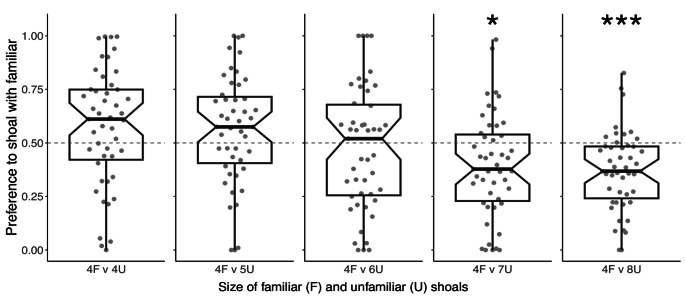
Notched box and whisker plots with overlaid raw data showing proportional time in the familiar shoaling zone relative to total time in both familiar and unfamiliar shoaling zones. Values of > 0.5 indicate that subjects spent more time in the familiar shoaling zone, while values of < 0.5 indicate they spent more time in the unfamiliar shoaling zone. Central lines represent medians, boxes extend from the 1st to the 3rd quartile, whiskers show the range up to 1.5x beyond the boxes, and the notches indicate approximate 95% confidence intervals for the medians

While subjects’ shoaling preferences also varied with the size of the unfamiliar shoal (Fig. 3), they did not show preferences for either shoal in the tests with four unfamiliar fish (mean ± SE shoaling preference score = 0.574 ± 0.040, t_46_ = 1.848, *p* = 0.355), 5 unfamiliar fish (mean = 0.563 ± 0.037, t_46_ = 1.692, *p* = 0.390), or 6 unfamiliar fish (mean = 0.474 ± 0.042, t_46_=−0.618, *p* = 0.978). Subjects had significant preferences for the larger unfamiliar shoal in tests with 7 unfamiliar fish (mean = 0.392 ± 0.035, t_46_=−3.057, *p* = 0.026) and 8 unfamiliar fish (mean = 0.370 ± 0.027, t_46_=−4.836, *p* < 0.001).

## Discussion

Individual zebrafish spent significantly more time interacting with familiar than unfamiliar conspecifics when both shoals consisted of four stimulus fish, replicating previous findings reporting preferences for familiarity in zebrafish (Gerlach and Lysiak 2006; Mukherjee and Bhat 2023; Swaney et al. 2024). However, we found that the preferences of zebrafish for larger shoals (Seguin and Gerlai 2017) took priority over familiarity preferences in the choice tests with the largest unfamiliar shoals of 7 or 8 individuals, but that when unfamiliar shoals were only slightly bigger (5 or 6 individuals) than the familiar shoals, subjects did not show clear preferences for either shoal. Across the five choice tests, we saw a gradual shift in preference according to the relative difference in shoal sizes, indicating that zebrafish are able to integrate information about both familiarity and shoal size in their shoaling decisions, and that they weighed the relative size of the unfamiliar shoal against that of the familiar shoal in their decisions over which group to associate with. In contrast to our results, the shoaling cichlid *Lamprologus callipterus* is able to discriminate between shoals based on familiarity, but their preferences for larger shoals are unaffected by the familiarity status of those shoals (Durrer et al. 2020).

The ability of zebrafish to integrate different group properties into shoaling decisions has also been shown in other contexts. Zebrafish prefer active shoals to slow-moving ones, but when this conflicts with the preference for large shoals, they shift their preference depending on the relative size difference between the active and inactive shoals (Pritchard et al. 2001). The ability of fish to combine information from multiple sources into behavioural decisions has also been documented in other contexts (Ward and Mehner 2010), however not all social fish appear able to integrate multiple group properties into shoaling decisions. Killifish choosing between shoals that vary in body size and also in shoal size have been shown to preferentially shoal with similarly-sized individuals even if the shoal of dissimilar individuals is numerically larger (Krause and Godin 1994). This suggests that they have a hierarchy of shoaling preferences, and that body size similarity is more important than shoal size in shoaling decisions. Female swordtails prefer large shoals to small ones, and also prefer to associate with similarly-sized individuals over dissimilarly-sized ones, however they do not exhibit any shoaling preferences when choosing between groups where these preferences are in conflict with each other (Wong and Rosenthal 2005).

Although we did not explicitly set out to test numerosity, our results are relevant when considering the ability of zebrafish to discriminate quantities. Researchers have often used shoal size preferences as a basis for measuring counting abilities in social fish by testing preferences for large shoals in choice tests (Agrillo and Bisazza 2018). While shoal size discrimination has been demonstrated from early ages in zebrafish (Sheardown et al. 2022), mixed results have been reported for adults, with larger shoals not preferred if the difference in size between two shoals is relatively small (Potrich et al. 2015), or if the total number of zebrafish in two stimulus shoals is too large (Seguin and Gerlai 2017). However, our results indicate that in choice tests involving both size and familiarity, adult zebrafish were able to distinguish shoals that differ only slightly in size, as familiarity preferences were seen when shoals were equal in size, but not when the unfamiliar shoals were only slightly larger in the tests of 4 versus 5 individuals. Similarly, in tests with stimulus shoals of 4 versus 8 individuals, subjects clearly distinguished between the shoals despite the large total number of stimulus fish in these tests, suggesting that this is not necessarily a barrier to shoal size estimation. The types of stimulus shoals we used may have played a role in these differing results, as choosing between shoals that varied in two group properties may have provided additional information which enabled subjects to discriminate between the shoals when they would not be able to do so if deciding solely on the basis of group sizes. It is also possible that other factors may have contributed, as shoaling decisions and numerosity could be affected by the documented genetic variation between captive populations (Suurväli et al. 2019), while zebrafish shoaling behaviour has also been shown to be sensitive to subtle changes in test design (Swaney et al. 2024).

An interesting question raised by our results is whether the preferences we observed for familiar shoals and for larger unfamiliar shoals are indicative of the adaptive value of those groups, and the benefits that individuals might gain through association. Studies have shown that individuals in larger shoals experience reduced predation risk (Krause and Godin 1995; Polyakov et al. 2022), and that predators are less likely to attack shoals that are more cohesive and coordinated (Ioannou et al. 2012), properties associated with familiarity (Lucon-Xiccato et al. 2022). Foraging success has also been shown to be higher for both familiar shoals (Morrell et al. 2008) and larger shoals (Day et al. 2001; Hintz and Lonzarich 2018). Given such benefits from both familiar and larger shoals, it would be revealing to test how much the preferences for shoals that vary in these dimensions correspond with the benefits gained by individuals in such shoals. Although potentially challenging to investigate, this is a particularly interesting question as it would show whether, and to what degree, individual fish make rational choices (Petrillo and Rosati 2021) when deciding between two shoals, and whether their evaluation of social groups accurately reflects the benefits provided by those groups.

## Data Availability

The data generated and analysed in this study, and the R code used to analyse them, will be available on publication in the Zenodo repository (10.5281/zenodo.15625544).
